# Cost-effectiveness of anti-SARS-CoV-2 antibody diagnostic tests in Brazil

**DOI:** 10.1371/journal.pone.0264159

**Published:** 2022-02-25

**Authors:** Tália Santana Machado de Assis, Mariana Lourenço Freire, Janaína de Pina Carvalho, Ana Rabello, Gláucia Cota

**Affiliations:** 1 Centro Federal de Educação Tecnológica de Minas Gerais, Contagem, Minas Gerais, Brazil; 2 Pesquisa Clínica e Políticas Públicas em Doenças Infecto-Parasitárias, Instituto René Rachou, Fundação Oswaldo Cruz, Belo Horizonte, Minas Gerais, Brazil; Massachusetts General Hospital, UNITED STATES

## Abstract

**Background:**

Although serologic tests for COVID-19 diagnosis are rarely indicated nowadays, they remain commercially available and widely used in Brazil. The objective of this study was to evaluate the cost-effectiveness of anti-SARS-CoV-2antibody diagnostic tests for COVID-19 in Brazil.

**Methods:**

Eleven commercially available diagnostic tests, comprising five lateral-flow immunochromatographic assays (LFAs) and six immunoenzymatic assays (ELISA) were analyzed from the perspective of the Brazilian Unified Health System.

**Results:**

The direct costs of LFAs ranged from US$ 11.42 to US$ 17.41and of ELISAs, from US$ 6.59 to US$ 10.31. Considering an estimated disease prevalence between 5% and 10%, the anti-SARS-CoV-2 ELISA (IgG) was the most cost-effective test, followed by the rapid One Step COVID-19 Test, at an incremental cost-effectiveness ratio of US$ 2.52 and US$ 1.26 per properly diagnosed case, respectively. Considering only the LFAs, at the same prevalence estimates, two tests, the COVID-19 IgG/IgM and the One Step COVID-19 Test, showed high effectiveness at similar costs. For situations where the estimated probability of disease is 50%, the LFAs are more costly and less effective alternatives.

**Conclusions:**

Nowadays there are few indications for the use of serologic tests in the diagnosis of COVID-19 and numerous commercially available tests, with marked differences are observed among them. In general, LFA tests are more cost-effective for estimated low-COVID-19-prevalences, while ELISAs are more cost-effective for high-pretest-probability scenarios.

## Introduction

The acute respiratory disease COVID-19, caused by the coronavirus SARS-CoV-2, had already reached 216 countries and surpassed 209,000,000 confirmed cases untilAugust21, 2021 [1]. In Brazil, since its introduction, more than 20 million cases and 574 thousand deaths had been reported [2]. One of the major impacts generated by the spread of SARS-CoV-2 is mainly related to the high number of patients requiring medical assistance in a short time, overloading the health systems causing it to collapse. In this sense, several public health measures have been employed to contain the transmission, such as the social distancing, quarantine of the infected patients and the massive testing of the population, representing crucial strategies for mitigating the spread of the disease [3].

The prompt diagnosis is the most effective way to disease control, containing its spread and preventing deaths. Reverse transcription–polymerase chain reaction (RT-PCR) is considered the gold standard for diagnosing acute-phase disease [4]. This technique requires complex laboratory infrastructure, specialized professionals and long time to the results, great challenges in limited resources settings. On the other hand, antibody-based tests hold a complementary role in the diagnostic of disease in negative RT-PCR symptomatic patients, several days after the onset of symptoms or on settings where the molecular test is not largely available [5]. The anti-SARS-CoV-2 antibody detection tests, include lateral-flow immunochromatographic assays (LFAs) and enzyme-linked immunosorbent assays (ELISAs), are also valued tools for epidemiological surveys, assisting in understanding of the disease spreading, in the impact of containment measures and prioritizing groups for vaccination.

The number of anti-SARS-CoV-2 antibody detection tests available worldwide has increased rapidly since the arise of the pandemic [6, 7]. In Brazil, given the impossibility of offering large-scale molecular tests, serological tests were largely used to diagnose COVID-19 during the first phase of the pandemic in 2020, and its use remains [8, 9]. Currently, serologic tests are rarely indicated in clinical practice but could still be used in epidemiological surveys. Cota et al (2020) evaluated the performance of these serological testes and reported higher sensitivity rate for patients after14 days onset of symptom and with severe clinical forms [10]. These results confirmed that tests based on antibody detection are not a good strategy for confirming acute COVID-19 cases and, if used for diagnosis purposes, it would be during the convalescent phase. On the other hand, and based on these results, a potential use of serological tests would be the epidemiological assessment of the spread of the infection. Regardless of the intend use for a diagnostic tool, in addition to accuracy, an economic analysis is a necessary step before the decision to incorporate a test, mainly in scenarios with limited resources and large number of available commercial options. It is especially important in current Brazilian context, marked by great social inequalities and progressive reductions in health and research budgets in recent years, which have further limited the response capacity to health crisis, such as caused by COVID-19. In this sense, this study aimed to support health managers providing information on cost-effectiveness of anti-SARS-CoV-2 antibody diagnostic tests for COVID-19 previously evaluated by Cota et al. (2020) [10].

## Methods

The study was conducted rigorously following recommendations of the Brazilian methodological guidelines for carrying out economic studies [11] and the information presented following recommendations of the Consolidated Health Economic Evaluation Reporting Standards (CHEERS) statement [12].

### Study design

The cost-effectiveness analysis for eleven anti-SARS-CoV-2 antibody diagnostic tests for COVID-19 was performed using an analytical decision model based on decision trees developed in TreeAge Pro 2015 software (TreeAge Software, Inc., Massachusetts, United States). The outcome of interest is a COVID-19 case properly diagnosed. Information subsequently to the COVID-19 diagnosis is limited, especially from the perspective of society, which limits the assessment of other outcomes such as impact on quality of life.

The analysis was performed under the perspective of the Brazilian public health system (SUS), which corresponds to the main payer of health costs in Brazil, where 75% of the population uses the public health system, a universal right guaranteed by law [13].

The target population are adult patients clinically suspected of COVID-19 after 14 days of symptoms onset, admitted at an outpatient or inpatient health care service. The analytical time was assumed as the interval from the clinical suspicion of COVID-19 and serological test request until the outcome assessment, in this case, the test result. This analytical time was defined as sufficiently large to capture all the costs and the target outcome involved during this serological diagnostic approach.

### Diagnostic tests

Eleven commercially availableanti-SARS-CoV-2 antibody tests for the diagnosis of COVID-19 registered in the Brazilian National Health Surveillance Agency (Agência Nacional de Vigilância Sanitária—ANVISA) were included in the analyses, five LFAs and six ELISAs (Table 1). The inclusion of these tests in the present economic analysis considered the availability of performance data obtained in Brazil [10], commercial availability, and the ability to obtain the costs of tests from manufacturers/distributors in Brazil.

**Table 1:** Performance of the evaluated COVID-19 diagnostic tests performed after 14 days from onset of symptoms.

Diagnostic tests	Sensitivity(%) 95% CI	Specificity(%) 95% CI
**Rapid Tests**		
One Step COVID- 19 Test (Guangzhou Wondfo Biotech Co., Ltd.)	83.7 (73.7–90.4)	100 (96.8–100)
COVID-19 IgG/IgM ECO Test (Eco Diagnostica Ltda)	89.2 (80.1–94.4)	99.1 (95.2–99.8)
COVID-19 IgG/IgM (Qingdao Hightop Biotech CO., Ltd.)	68.9 (57.6–78.3)	100 (96.8–100)
Imuno-RápidoCOVID-19 IgG/IgM (Wama produtos para laboratorio LTDA)	86.5 (76.9–92.5)	97.4 (92.6–99.1)
COVID-19 IgG/IgM (Gold Analisa Diagnóstica LTDA)	74.3 (63.3–82.9)	98.3 (93.9–99.6)
**ELISAs**		
COVID-19 ELISA IgM + IgA (VircellMicrobiologists)	95.9 (88.7–98.6)	23.3 (16.5–31.8)
COVID-19 ELISA IgG (Vircell Microbiologists)	93.2 (85.1–97.1)	53.4 (47.4–59.3)
Anti-SARS-CoV-2 ELISA (IgA) (Euroimmun Medicina Diagnóstica LTDA)	89.4 (77.4–95.4)	82.2 (72.7–89.8)
Anti-SARS-CoV-2 ELISA (IgG) (Euroimmun Medicina Diagnóstica LTDA)	87.2 (74.8–93.9)	95.8 (88.5–98.5)
Allserum EIA COVID19 IgM (MbiologDiagnosticos LTDA)	50.7 (39.4–62.0)	70.4 (61.2–78.2)
Allserum EIA COVID19 IgG (MbiologDiagnosticos LTDA)	78.9 (68.1–86.8)	98.1 (93.4–99.5)

Source: Cota et al., 2020 [9].

### Effectiveness

In the present economic analysis, the performance measure used was accuracy, defined by the sum of the corrected results, or the number of true positives plus true negatives, divided by the total number of tests performed. Accuracy rate was derived from sensitivity and specificity, as previously determined by Cota et al. [9]. It is the unique study performed in Brazil that assessed comparatively (in parallel) the clinical performance of serological tests available for diagnosing SARS-CoV-2. The study was conducted using a well-characterized panel of 289 serum samples, of which 173 were from patients with confirmation of SARS-CoV-2 infection by PCR in a nasopharyngeal swab (cases) and 116 samples from patients with serological markers for other infectious diseases (controls), such as dengue, Zika, Chagas disease, syphilis, toxoplasmosis, viral hepatitis, malaria, visceral leishmaniasis, cytomegalovirus, Epstein Barr virus infection, and HIV infection. Among the COVID-19 cases, 25 (15%) had up to 6 days of symptoms, 74 (43%) had between 7 and 14 days from the onset of symptoms, and 74 had 15 days or more from the onset of symptoms. Among this latter group, 19 patients (26%) had between 31 and 60 days from the onset of symptoms and 13 (17.5%) had more than 2 months from the onset of symptoms. Fifty-nine percent of patients met the criteria for acute respiratory distress syndrome. The performance of each diagnostic tests performed after 14 days from onset of symptoms is presented in Table 1.

### Direct costs

Direct costs were estimated by micro costing, a cost estimation method that involves direct enumeration of the cost of each resource required for a given health intervention [14, 15]. These estimates were performed in parallel to the performance evaluation of the diagnostic tests performed by Cota et al. [10]. The unit cost of each test was obtained from the test manufacturer/distributor in Brazil. The remuneration of health workers was obtained from the current Remuneration Table of the Municipality of Belo Horizonte, Minas Gerais, considering that the tests are performed by a laboratory technician [16]. The cost of safety and consumable materials was obtained from the Health Prices Bank of the Ministry of Health [17]. The equipment maintenance cost was obtained from the Contracts Sector of René Rachou Institute, Oswaldo Cruz Foundation. For the ELISAs, we assumed that in each reaction, the plate was used at full capacity. Details about the cost estimates are presented in the S1 Table. The year of analysis was 2020 and costs identified in other years were adjusted according to the Broad National Consumer Price Index (IPCA) cumulative for June, 2020. All costs were estimated in the Brazilian currency (reais) (R$) and then converted into US dollars (US$) (July 2020 month average commercial exchange rate for purchase: R$ 5.2796 = US$ 1.00) [18].

### Cost-effectiveness analytical model

The cost-effectiveness analyses were conducted separately for the two diagnostic modalities (LFA and ELISA), in addition to the global analysis considering all tests. For each analysis, three pretest probability scenarios were considered for COVID-19: 5%, 10%, and 50%. In this study, the effectiveness of diagnostic tests for COVID-19 was measured in terms of case properly diagnosed.

The analysis starts with a COVID-19 suspected patient being tested and ended with the result of the diagnostic test, which can be true positive, false negative, true negative, or false positive. Branches of the trees that ended with correct results (true positive or true negative) were given a value of 1 in the terminal nodes, and those that ended with incorrect results (false negative or false positive), i.e., those in which the test failed, were given a value of 0. S1 Fig illustrates the structure of the decision trees adopted.

First, the diagnostic tests were ranked from least expensive to most expensive. The test placed in the first row of the cost-effectiveness analysis table of results was the least expensive test and was the baseline comparator of this analysis. Based on this comparator, a new diagnostic alternative that had an increase in cost along with a reduction in effectiveness was termed an “absolutely dominated” strategy and was excluded from the analysis. The diagnostic alternative in which an increase in cost was associated with increased effectiveness was called "nondominated". For these diagnostic alternatives, the incremental cost-effectiveness ratio (ICER) was calculated, which is the ratio of the difference in costs of the diagnostic tests over the difference in their effectiveness, express in cost per correctly diagnosed case.

### Sensitivity analysis

The influence of uncertainties due to the variability in the parameters included in the model–sensitivity, specificity, and costs–was evaluated using deterministic univariate sensitivity analysis. In the deterministic sensitivity analysis, the values used for the variations in test performance were based on the 95% confidence intervals (95% CI) of the sensitivity and specificity rates estimated by Cota et al. [10]. The direct costs of the tests were also arbitrarily varied by ± 25% for this analysis.

## Results

For the rapid tests, the direct costs ranged from US$ 11.42 to US$ 17.41 (Table 2). For the ELISAs, from US$ 6.59 to US$ 10.31 (Table 3). The most significant cost component for both LFAs and ELISAs was the unit value of the diagnostic tests.

**Table 2:** Detailed cost of the items included in the direct cost estimates of the evaluated rapid tests for COVID-19 in Brazil, base year 2020.

Diagnostic test	Items included in the cost estimate	Value (US$)
One Step COVID-19 Test (Guangzhou Wondfo Biotech Co., Ltd.)	Unit cost of the test	9.47
	Laboratory technician remuneration	1.50
	Personal protective equipment	0.16
	Consumables	0.25
	Equipment maintenance	0.06
	**Total**	**11.44**
	**± 25% variation**	**8.58–14.29**
COVID-19 IgG/IgM ECO Test (Eco Diagnostica Ltda)	Unit cost of the test	15.63
	Laboratory technician remuneration	1.32
	Personal protective equipment	0.16
	Consumables	0.25
	Equipment maintenance	0.06
	**Total**	**17.41**
	**± 25% variation**	**13.06–21.76**
COVID-19 IgG/IgM (Qingdao Hightop Biotech CO., Ltd.)	Unit cost of the test	9.45
	Laboratory technician remuneration	1.50
	Personal protective equipment	0.16
	Consumables	0.25
	Equipment maintenance	0.06
	**Total**	**11.42**
	**± 25% variation**	**8.56–14.27**
Imuno-RápidoCOVID-19 IgG/IgM (Wama produtos para laboratorio LTDA)	Unit cost of the test	14.21
	Laboratory technician remuneration	1.50
	Personal protective equipment	0.16
	Consumables	0.25
	Equipment maintenance	0.06
	**Total**	**16.17**
	**± 25% variation**	**12.13–20.22**
COVID-19 IgG/IgM (Gold Analisa Diagnóstica LTDA)	Unit cost of the test	12.31
	Laboratory technician remuneration	1.50
	Personal protective equipment	0.16
	Consumables	0.25
	Equipment maintenance	0.06
	**Total**	**14.28**
	**± 25% variation**	**10.71–17.85**

**Table 3:** Detailed cost of the items included in the direct cost estimates of the evaluated immunoenzymatic assays for COVID-19 in Brazil, base year 2020.

Diagnostic test	Items included in the cost estimate	Value (US$)
COVID-19 ELISA IgM + IgA (VircellMicrobiologists)	Unit cost of the test	5.21
	Laboratory technician remuneration	0.80
	Personal protective equipment	0.15
	Consumables	0.27
	Equipment maintenance	0.15
	**Total**	**6.59**
	**± 25% variation**	**4.95–8.24**
COVID-19 ELISA IgG (Vircell Microbiologists)	Unit cost of the test	5.21
	Laboratory technician remuneration	0.80
	Personal protective equipment	0.15
	Consumables	0.30
	Equipment maintenance	0.15
	**Total**	**6.62**
	**± 25% variation**	**4.97–8.28**
Anti-SARS-CoV-2 ELISA (IgA) or Anti-SARS-CoV-2 ELISA (IgG) (Euroimmun Medicina Diagnóstica LTDA)	Unit cost of the test	5.70
	Laboratory technician remuneration	0.80
	Personal protective equipment	0.15
	Consumables	0.30
	Equipment maintenance	0.15
	**Total**	**7.12**
	**± 25% variation**	**5.34–8.90**
Allserum EIA COVID19 IgM or Allserum EIA COVID19 IgG (Mbiolog Diagnosticos LTDA)	Unit cost of the test	8.89
	Laboratory technician remuneration	0.80
	Personal protective equipment	0.15
	Consumables	0.30
	Equipment maintenance	0.15
	**Total**	**10.31**
	**± 25% variation**	**7.67–12.89**

The cost-effectiveness analyses for the LFAs and ELISAs, considering the hypothetical pretest probability scenarios of 5%, 10%, and 50%, are shown in Table 4.

**Table 4:** Cost-effectiveness analysis of rapid tests and immunoenzymatic assays for three pretest probability scenarios of 5%, 10%, and 50%.

Diagnostic tests	C (US$)	IC (US$)	Prevalence of 5%				Prevalence of 10%				Prevalence of 50%			
			E	IE	ICER / CDC (US$)	DM	E	EI	ICER/CDC (US$)	DM	E	EI	ICER / CDC (US$)	DM
**Rapid tests**														
COVID-19 IgG/IgM (Qingdao Hightop Ltd.)	11.42	**-**	0.98	**-**	**-**	ND	0.97	**-**	**-**	ND	0.84	**-**	**-**	ND
One Step COVID- 19 Test (Guangzhou Wondfo Ltd.)	11.44	0.02	0.99	0.01	2.52	ND	0.98	0.02	1.26	ND	0.92	0.07	0.25	ND
COVID-19 IgG/IgM (Gold Analisa LTDA)	14.28	2.84	0.97	-0.02	*	AbD	0.96	-0.03	*	AbD	0.86	-0.06	*	AbD
Imuno-RápidoCOVID-19 IgG/IgM (Wama LTDA)	16.17	4.74	0.96	-0.03	*	AbD	0.96	-0.02	*	AbD	0.92	0.00	*	AbD
COVID-19 IgG/IgM ECO (Eco Diagnostica Ltda)	17.41	1.24	0.98	-0.01	*	AbD	0.98	-0.00	*	AbD	0.94	0.02	298.60	ND
**Immunoenzymatic assays**														
COVID-19 ELISA IgM+IgA (VircellMicrobiologists)	6.59	-	0.27	-	-	ND	0.30	-	-	ND	0.60	-	-	ND
COVID-19 ELISA IgG (Vircell Microbiologists)	6.62	0.03	0.55	0.28	0.11	ND	0.57	0.27	0.11	ND	0.73	0.14	0.23	ND
Anti-SARS-CoV-2 ELISA (IgA) (Euroimmun LTDA)	7.12	0.49	0.82	0.27	1.81	ND	0.83	0.26	1.92	ND	0.86	0.12	3.95	ND
Anti-SARS-CoV-2 ELISA (IgG) (Euroimmun LTDA)	7.12	0.00	0.96	0.13	0.00	ND	0.95	0.12	0.00	ND	0.92	0.06	0.00	ND
Allserum EIA COVID19 IgM (MbiologDiagnosticos LTDA)	10.31	3.19	0.69	-0.27	*	AbD	0.68	-0.27	*	AbD	0.60	-0.31	*	AbD
Allserum EIA COVID19 IgG (MbiologDiagnosticos LTDA)	10.31	3.19	0.97	0.02	212.77	ND	0.96	0.01	319.15	ND	0.88	-0.03	*	AbD

C: cost; IC: incremental cost; E: effectiveness; IE: incremental effectiveness; ICER/CDC: incremental cost-effectiveness ratioper correctly diagnosed case; DM: dominance; ND: nondominated; AbD: absolutely dominated

*negative ICER.

The comparison among LFAs was performed considering a hospital admission screening scenario and places lacking laboratory infrastructure, where point-of-care tests are the indicated method. Among these rapid tests, COVID-19 IgG/IgM (Qingdao Hightop Biotech CO., Ltd.) had the lowest cost (US$ 11.42) and an effectiveness of 0.98 and 0.97 under an assumption of 5% and 10% pretest probability, respectively. The second-lowest cost was for the One Step COVID-19 Test (Guangzhou Wondfo Biotech Co., Ltd.), with a slight increase in effectiveness at an ICER of US$ 2.52 and US$ 1.26per correctly diagnosed case for the pretest probabilities of 5% and 10%, respectively. For the other LFAs, the increase in cost was not accompanied by an increase in effectiveness. Conversely, for a pretest probability of 50%, two tests showed a marked increase in effectiveness compared to the cheapest test: One Step COVID-19 Test (GuangZhouWondfo Biotech Co., Ltd.) and COVID-19 IgG/IgM ECO (Eco Diagnostica Ltda), with respective ICER values of US$ 0.25 and US$ 298.60per correctly diagnosed case. As both tests achieved similar effectiveness, the option with the lower ICER, the One Step COVID-19 Test, became the most cost-effective alternative.

For health services with sufficient laboratory infrastructure, ELISA tests, which have higher sensitivity and lower cost, would be the indicated option. In the comparison of ELISAs, the test from Vircell Microbiologists for detection of IgM+IgA was the one with the lowest cost (US$ 6.59), and it had a low effectiveness, ranging from 0.27 to 0.60% in the different pretest probability scenarios. In turn, the same manufacturer’s test for IgG detection, for the pretest probabilities of 5%, 10%, and 50%, showed ICERs equal to US$ 0.11, US$ 0.11 and US$ 0.23per correctly diagnosed case, respectively. This test had a slightly higher increase in effectiveness, ranging from 0.55 to 0.73. The IgG ELISA from Mbiolog Diagnosticos LTDA also had a higher effectiveness for the scenarios of 5% and 10% pretest probability, but associated with a high ICER, at US$ 212.77 and US$ 319.15per correctly diagnosed case, respectively. The Euroimmun Medicina Diagnóstica LTDA test for IgG detection was more cost-effective in all evaluated pretest probability scenarios.

Table 5 shows the results of the cost-effectiveness analyses considering all diagnostic tests, regardless of the platform (LFA and ELISA), and the hypothetical disease probability scenarios of 5%, 10%, and 50%. In all evaluated scenarios, the anti-SARS-CoV-2 ELISA (IgG) (Euroimmun LTDA) showed the lowest ICER, with effectiveness ranging from 0.92 to 0.96%. Among the LFAs, the One Step COVID-19 Test (Guangzhou Wondfo Ltd.) was the only one that was not dominated in all prevalence scenarios, with effectiveness ranging from 0.92 to 0.99% per properly diagnosed case. However, under the scenario of 50% pretest probability, the ICER was US$ 863.70per correctly diagnosed case, with no increase in effectiveness compared to the ELISA.

**Table 5:** Cost-effectiveness analysis including all tests for three hypothetical pretest probability scenarios (5%, 10%, and 50%).

Diagnostic tests	C (US$)	IC (US$)	Prevalence of 5%				Prevalence of 10%				Prevalence of 50%			
			E	IE	ICER/CDC (US$)	DM	E	IE	ICER/CDC (US$)	DM	E	IE	ICER/CDC (US$)	DM
COVID-19 ELISA IgM+IgA (VircellMicrobiologists)	6.59	**-**	0.27	**-**	**-**	ND	0.30	**-**	**-**	ND	0.60	**-**	**-**	ND
COVID-19 ELISA IgG (Vircell Microbiologists)	6.62	0.03	0.55	0.28	0.11	ND	0.57	0.27	0.11	ND	0.73	0.14	0.23	ND
Anti-SARS-CoV-2 ELISA (IgA) (Euroimmun LTDA)	7.12	0.49	0.82	0.27	1.81	ND	0.83	0.26	1.92	ND	0.86	0.12	3.95	ND
Anti-SARS-CoV-2 ELISA (IgG) (Euroimmun LTDA)	7.12	0.00	0.96	0.13	0.00	ND	0.95	0.12	0.00	ND	0.92	0.06	0.00	ND
Allserum EIA COVID19 IgM (MbiologDiagnosticos LTDA)	10.31	3.19	0.69	-0.26	*	AbD	0.68	-0.27	*	AbD	0.60	-0.31	*	AbD
Allserum EIA COVID19 IgG (MbiologDiagnosticos LTDA)	10.31	3.19	0.97	0.02	212.77	Extd	0.96	0.01	319.15	Extd	0.88	-0.03	*	AbD
COVID-19 IgG/IgM (Qingdao Hightop Ltd.)	11.42	1.11	0.98	0.01	79.15	Extd	0.97	0.01	138.50	Extd	0.84	-0.07	*	AbD
One Step COVID- 19 Test (Guangzhou Wondfo Ltd.)	11.44	0.02	0.99	0.01	2.52	ND	0.98	0.02	1.26	ND	0.92	0.00	863.70	Extd
COVID-19 IgG/IgM (Gold Analisa LTDA)	14.26	2.84	0.97	-0.02	*	AbD	0.96	-0.03	*	AbD	0.86	-0.06	*	AbD
Imuno-RápidoCOVID-19 IgG/IgM (Wama LTDA)	16.17	4.74	0.96	-0.03	*	AbD	0.96	-0.02	*	AbD	0.92	0.00	*	AbD
COVID-19 IgG/IgM ECO (Eco Diagnostica Ltda)	17.41	5.97	0.98	-0.01	*	AbD	0.98	-0.00	*	AbD	0.94	0.02	298.60	ND

C: cost; IC: incremental cost; E: effectiveness; IE: incremental effectiveness; ICER/PCDC: incremental cost-effectiveness ratioper correctly diagnosed case; DM: dominance; ND: nondominated; AbD: absolutely dominated; Extd: extended dominance (weak).

Variation of costs, sensitivity and specificity of the LFAs and ELISAs influenced all results evaluated in the deterministic sensitivity analysis. Probabilistic sensitivity analyzes were not performed because Brazil does not have a defined willingness-to-pay threshold.

## Discussion

Besides the speed, accuracy, and simplicity of the diagnostic, the cost-effectiveness of SARS-CoV-2 diagnostic tests are important global concern, providing evidence-based solutions for the decision making [19, 20]. Recent analysis has shown that testing people with any COVID-19-consistent symptoms is more cost-effective than the restrictive use of the tests for severe patients [21]. In this sense, the combination of effective diagnostic tests with different applications is required as effective public health strategies [22, 23]. In low-resource settings with limited capacity to perform RT-PCR, different strategies to improve the diagnostic access has been analyzed and considered cost-effective, such as the use of antigen detection rapid diagnostic tests [24] and sample pooling method [25]. Although the limited use of serological tests routinely for COVID-19 diagnostic, these tests has been used in several scenarios, including as a diagnosis tool for negative RT-PCR patients with several days since the onset of symptoms. Considering differences among performance and costs, in addition to the large number of commercially available antibody detection tests, we performed a cost-effectivity analyzes using as assumptions in the model the accuracy data previously and locally stablished for serological tests performed after 14 days of the onset of symptoms [10]. Our results may assist health managers in decision-making regarding COVID-19, mainly about the use of serological tests.

Although serological tests are not routinely recommended in the diagnostic evaluation of COVID-19 suspected cases, due to its late positivity in the course of infection, these tests have been extensively used in Brazil for this purpose in public health services and in the private sector during the first months of the COVID-10 pandemic. [17, 18]. Another alleged use for serology would be the identification of IgG antibodies for SARS-CoV-2 as a proxy for acquired immunity and a “passport” for returning to activities, an unapproved use given the lack of confirmation of the immunity conferred by these antibodies [19] and increasing number of reports of reinfection in recent literature [20, 21]. Thus, while we emphasize the misuse of serological tests for the investigation of acute symptomatic cases, we reinforce the importance of carrying out the present analysis considering that these tests are widely commercialized and still in use in a scenario of inequality access to timely diagnosis in Brazil. Differences in performance and cost between assays based on the same platform, and the large number of tests made commercially available, make economic evaluations the ensuing and necessary step after accuracy analysis. In this sense, the use of cost-effectiveness analysis could support decisions in public health and are even more relevant in contexts of limited resources. However, this economic analysis applies exclusively to serology for the diagnosis of symptomatic patients after 14 days of the onset of symptoms, a period in which the best performance of the immunological tests has been confirmed and when tests based on the identification of the agent show very low sensitivity. Nevertheless, the inherent limitations of the serological method are undeniable, and need to be considered from the broader perspective of all possible diagnostic algorithms. In addition to the already mentioned late diagnosis, it is also worth mentioning the decreasing of specificity of the all serological tests along the progression of the COVID-19 epidemic curve, considering that more people are having contact with the SARS-CoV-2 virus and possibly acquiring antibodies, while other viral agents tend to return to circulate causing acute respiratory cases. That is, increasingly, a positive result will have less positive predictive value in the assessment of an acute respiratory episode. All these issues need to generate a deep debate on the relevance of maintaining some investment in these tests, which have the approval of health regulatory agencies in several countries are supported by a massive marketing strategy.

In the Brazilian scenario and considering these evaluated tests, the LFA have higher unit costs than the ELISA tests and are generally less sensitive but more specific than the latter. In the head-to-head comparison, the One Step COVID-19 Test (Guangzhou Wondfo Biotech Co, Ltd.), provided by the Brazilian Public Health System, was confirmed in all evaluated pretest probability scenarios as more cost-effective option among the LFAs included in this analysis. Among the ELISAs, the anti-SARS-CoV-2 ELISA (IgG) (Euroimmun LTDA) was the test with the highest effectiveness and lowest ICER.

In a hypothetical scenario with all available tests, under the three analyzed pretest probabilities, the Euroimmun ELISA test for IgG antibodies was the most cost-effective alternative. The only LFA that remained more cost-effective in all three pretest probability scenarios was the One-Step COVID-19 Test (Guangzhou Wondfo Biotech Co., Ltd.), but at the expense of a large cost increase per correctly diagnosed case, especially in a situation of high probability of disease, as in the case of hospitalized patients with symptoms suggestive of the disease. These findings confirm that in this context, the test based on ELISA is the best option. Our analyses show that a disease prevalence between 5% and 10%, a percentage that is estimated to be found to most Brazilian cities, does not alter the dominance relationship between the tests, which allows decision-making for acquisition and use of serological tests applicable to most outpatient health services, where mild cases are concentrated [26, 27]. Thus, for sites with a low prevalence of SARS-CoV-2 infection, the effectiveness of LFAs is higher than for ELISA tests, with an incremental cost that decreases as the prevalence increases. However, in clinical situations with a high probability of SARS-CoV-2 infection, such as hospitalized patients with various signs and symptoms suggestive of COVID-19, ELISA tests are more cost-effective. In all scenarios, tests based on IgA and IgM antibodies achieved only moderate effectiveness at a high additional cost, so these strategies had unfavorable cost-effectiveness. These observations confirm that health care decisions should be evaluated considering the conditions of real scenarios. There probably is no ideal test but rather a more appropriate test for a given situation.

Despite of being the first cost-effectiveness study assessing the serological tests for COVID-19 diagnosis in the context of Brazil, one of the world’s pandemic epicenters, this study has limitations. According to the study conducted by Cota et al. (2020) [10], which provided sensitivity and specificity rates for the tests addressed in this economic evaluation, the performance of the tests was higher among patients presenting severe clinical disease, compared to mild forms. Here, we assumed the average performance of the tests to avoid multiple models of analysis, which could hinder a more comprehensive conclusion. The perspective of the public health system adopted here can also generate difficulties in translating the conclusions into other scenarios, specifically when the payer is the private sector or the tests are performed in a facility different from a health care unit, such as a drugstore, for example. Finally, despite of being useful for comparing technologies and different studies, the establishment of an unique cost-effectiveness threshold, or a fixed value of willingness to pay is not universally accepted and remains in debate [28, 29]. The main reason is that it would be unable to capture all the important values for different countries and societies. Different from other countries, in Brazil there is no defined cost-effectiveness threshold for a new health technology be incorporated.

A strength of the present study is the use of performance data generated in a study conducted in Brazil. The cost estimate based on micro costing also approximates our estimates of the real cost of the intervention, which, added to the local performance data of the tests, confers reliability to the results and high applicability to the Brazilian scenario. Our observations, however, also indicate that antibody detection tests from different manufacturers may differ significantly in cost and performance. This study’s limitations include the existence of many commercially available tests in Brazil besides those included here. The performance of the LFAs was based on the results of tests performed in serum and not in capillary blood, which may have overestimated the performance of these tests when compared to the actual conditions of point-of-care use. Finally, because serological sensitivity is correlated with disease severity, the effectiveness of the evaluated tests could differ significantly in asymptomatic individuals. Thus, these observations should not be extrapolated to other contexts except in the diagnostic approach to patients with COVID-19 suspicion.

Economic studies evaluating diagnostic tests for COVID-19 were not found in the literature. Thus, the results presented here, although using data from the Brazilian reality, are novel, relevant, and potentially useful scientific evidence for health managers worldwide. Overall, the present analysis shows that despite the large number of available COVID-19 diagnostic tests, there are marked differences between them in addition to the differences in the test modality itself. Considering these aspects, the cost-effectiveness analyses are the indicate tool to assist the comparison among different interventions in the decision-making process. It is important to emphasize that in the current context, there are few indications for the use of serological strategy for the diagnosis of COVID-19. Despite of that, like any diagnostic approach, it requires besides performance data, individualized evaluation of the estimated disease probability and of the installed laboratorial infrastructure.

## Supporting information

S1 Fig Structure of the decision trees used. (PDF)

S1 Table Detailed costs of the items included in the direct cost estimates of the diagnostic test evaluated. ^1^Brazil. Diário Oficial do Município Lei n° 10.948, 13 de julho de 2016. vailable: http://portal6.pbh.gov.br/dom/iniciaEdicao.do?method=DetalheArtigo&pk=1165759; ^2^Brazil. Ministry of Health. SIGTAP—Sistema de Gerenciamento da Tabela de Procedimentos, Medicamentos e OPM do SUS. 2020. Available: http:/f/sigtap.datasus.gov.br/tabela-unificada/app/sec/inicio.jsp.
(PDF)
