# Supplementary figures and images for: Cost-effectiveness of anti-SARS-CoV-2 antibody diagnostic tests in Brazil
Source: PLoS One. 2022 Feb 25;17(2):e0264159. doi: 10.1371/journal.pone.0264159 (PMC8880880; doi:10.1371/journal.pone.0264159)

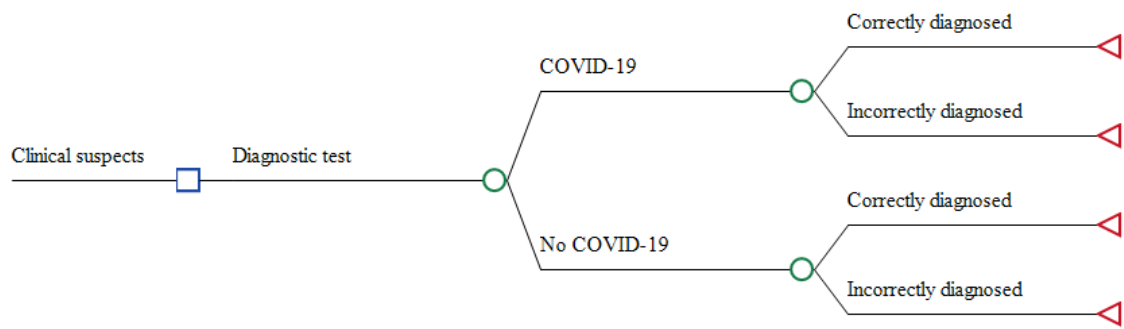

1

2 **Figure S1.** Structure of the decision trees used.

3

Supplement: S1 Fig — (PDF) [file pone.0264159.s001.pdf]
