# Supplementary material for: Cost-effectiveness of anti-SARS-CoV-2 antibody diagnostic tests in Brazil
Source: PLoS One. 2022 Feb 25;17(2):e0264159. doi: 10.1371/journal.pone.0264159 (PMC8880880; doi:10.1371/journal.pone.0264159)
Supplement: S1 Table — 1Brazil. Diário Oficial do Município Lei n° 10.948, 13 de julho de 2016. vailable: http://portal6.pbh.gov.br/dom/iniciaEdicao.do?method=DetalheArtigo&pk=1165759; 2Brazil. Ministry of Health. SIGTAP—Sistema de Gerenciamento da Tabela de Procedimentos, Medicamentos e OPM do SUS. 2020. Available: http:/f/sigtap.datasus.gov.br/tabela-unificada/app/sec/inicio.jsp. (PDF) [file pone.0264159.s002.pdf]

S2 Table. Detailed costs of the items included in the direct cost estimates of the diagnostic test evaluated.

Diagnostic test	Items included in the cost estimate	Description and Quantity	Cost (US$)	Data sources
One Step COVID-19 Test (Guangzhou Wondfo Biotech Co., Ltd.)	Unit value of the test	1 test	9.47	Test manufacturer/distributor in Brazil
	Laboratory technician remuneration (40h a week)	20 min to collected biological material + 21 min to performing the test	1.50	The salaries of health professionals of the municipality of Belo Horizonte, Minas Gerais, Brazil[1]
	Personal protective equipment	4 gloves and 2 masks	0.16	Brazilian Health System Reimbursement Values Table[2]
	Consumables	Alcohol solution (10mL), cotton (5g), needle (1u), syringe (1u), and blood collection tubes (1u)	0.25	Brazilian Health System Reimbursement Values Table[2]
	Equipment maintenance	Maintenance of centrifuge/patient	0.06	The business contract sector of one Brazilian health institute
	**Total**		**11.44**	
COVID-19 IgG/IgM ECO Test (Eco Diagnostica Ltda)	Unit value of the test	1 test	15.63	Test manufacturer/distributor in Brazil
	Laboratory technician remuneration (40h a week)	20 min to collected biological material + 16 min to performing the test	1.32	The salaries of health professionals of the municipality of Belo Horizonte, Minas Gerais, Brazil[1]
	Personal protective equipment	4 gloves and 2 masks	0.16	Brazilian Health System Reimbursement Values Table[2]

	Consumables	Alcohol solution (10mL), cotton (5g), needle (1u), syringe (1u), and blood collection tubes (1u)	0.25	Brazilian Health System Reimbursement Values Table[2]
	Equipment maintenance	Maintenance of centrifuge/patient	0.06	The business contract sector of one Brazilian health institute
	**Total**		**17.41**	
COVID-19 IgG/IgM (Qingdao Hightop Biotech CO., Ltd.)	Unit value of the test	1 test	9.45	Test manufacturer/distributor in Brazil
	Laboratory technician remuneration (40h a week)	20 min to collected biological material + 21 min to performing the test	1.50	The salaries of health professionals of the municipality of Belo Horizonte, Minas Gerais, Brazil[1]
	Personal protective equipment	4 gloves and 2 masks	0.16	Brazilian Health System Reimbursement Values Table[2]
	Consumables	Alcohol solution (10mL), cotton (5g), needle (1u), syringe (1u), and blood collection tubes (1u)	0.25	Brazilian Health System Reimbursement Values Table[2]
	Equipment maintenance	Maintenance of centrifuge/patient	0.06	The business contract sector of one Brazilian health institute
	**Total**		**11.42**	
Imuno-Rápido COVID-19 IgG/IgM (Wama produtos para laboratorio LTDA)	Unit value of the test	1 test	14.21	Test manufacturer/distributor in Brazil
	Laboratory technician remuneration (40h a week)	20 min to collected biological material + 21 min to performing the test	1.50	The salaries of health professionals of the municipality of Belo Horizonte, Minas Gerais, Brazil[1]
	Personal protective equipment	4 gloves and 2 masks	0.16	Brazilian Health System Reimbursement Values Table[2]

	Consumables	Alcohol solution (10mL), cotton (5g), needle (1u), syringe (1u), and blood collection tubes (1u)	0.25	Brazilian Health System Reimbursement Values Table[2]
	Equipment maintenance	Maintenance of centrifuge/patient	0.06	The business contract sector of one Brazilian health institute
	**Total**		**16.17**	
COVID-19 IgG/IgM (Gold Analisa Diagnóstica LTDA)	Unit value of the test	1 test	12.31	Test manufacturer/distributor in Brazil
	Laboratory technician remuneration (40h a week)	20 min to collected biological material + 21 min to performing the test	1.50	The salaries of health professionals of the municipality of Belo Horizonte, Minas Gerais, Brazil[1]
	Personal protective equipment	4 gloves and 2 masks	0.16	Brazilian Health System Reimbursement Values Table[2]
	Consumables	Alcohol solution (10mL), cotton (5g), needle (1u), syringe (1u), and blood collection tubes (1u)	0.25	Brazilian Health System Reimbursement Values Table[2]
	Equipment maintenance	Maintenance of centrifuge/patient	0.06	The business contract sector of one Brazilian health institute
	**Total**		**14.28**	
COVID-19 ELISA IgM + IgA (Vircell Microbiologists)	Unit value of the test	1 test	5.21	Test manufacturer/distributor in Brazil
	Laboratory technician remuneration (40h a week)	20 min to collected biological material + 2 min to performing the test	0.80	The salaries of health professionals of the municipality of Belo Horizonte, Minas Gerais, Brazil[1]
	Personal protective equipment	4 gloves and 2 masks	0.15	Brazilian Health System Reimbursement Values Table[2]

	Consumables	Alcohol solution (10mL), cotton (5g), needle (1u), syringe (1u), and blood collection tubes (1u), sterilized filter tips (2u), 96-well plate (1u)	0.27	Brazilian Health System Reimbursement Values Table[2]
	Equipment maintenance	Maintenance of centrifuge/patient, pipettes calibration and refrigerator	0.15	Test manufacturer/distributor in Brazil
	**Total**		**6.59**	
COVID-19 ELISA IgG (Vircell Microbiologists)	Unit value of the test	1 test	5.21	Test manufacturer/distributor in Brazil
	Laboratory technician remuneration (40h a week)	20 min to collected biological material + 2 min to performing the test	0.80	The salaries of health professionals of the municipality of Belo Horizonte, Minas Gerais, Brazil[1]
	Personal protective equipment	4 gloves and 2 masks	0.15	Brazilian Health System Reimbursement Values Table[2]
	Consumables	Alcohol solution (10mL), cotton (5g), needle (1u), syringe (1u), and blood collection tubes (1u), sterilized filter tips (3u), 96-well plate (1u)	0.30	Brazilian Health System Reimbursement Values Table[2]
	Equipment maintenance	Maintenance of centrifuge/patient, pipettes calibration and refrigerator	0.15	The business contract sector of one Brazilian health institute
	**Total**		**6.62**	
Anti-SARS-CoV-2 ELISA (IgA) or Anti-SARS-CoV-2 ELISA (IgG) (Euroimmun Medicina Diagnóstica LTDA)	Unit value of the test	1 test	5.70	Test manufacturer/distributor in Brazil
	Laboratory technician remuneration (40h a week)	20 min to collected biological material + 2 min to performing the test	0.80	The salaries of health professionals of the municipality of Belo Horizonte, Minas Gerais, Brazil[1]

	Personal protective equipment	4 gloves and 2 masks	0.15	Brazilian Health System Reimbursement Values Table[2]
	Consumables	Alcohol solution (10mL), cotton (5g), needle (1u), syringe (1u), and blood collection tubes (1u), sterilized filter tips (3u), 96-well plate (1u)	0.30	Brazilian Health System Reimbursement Values Table[2]
	Equipment maintenance	Maintenance of centrifuge/patient, pipettes calibration and refrigerator	0.15	The business contract sector of one Brazilian health institute
	**Total**		**7.12**	
Allserum EIA COVID19 IgM or Allserum EIA COVID19 IgG (Mbiolog Diagnosticos LTDA)	Unit value of the test	1 test	8.89	Test manufacturer/distributor in Brazil
	Laboratory technician remuneration (40h a week)	20 min to collected biological material + 2 min to performing the test	0.80	The salaries of health professionals of the municipality of Belo Horizonte, Minas Gerais, Brazil[1]
	Personal protective equipment	4 gloves and 2 masks	0.15	Brazilian Health System Reimbursement Values Table[2]
	Consumables	Alcohol solution (10mL), cotton (5g), needle (1u), syringe (1u), and blood collection tubes (1u), sterilized filter tips (3u), 96-well plate (1u)	0.30	Brazilian Health System Reimbursement Values Table[2]
	Equipment maintenance	Maintenance of centrifuge/patient, pipettes calibration and refrigerator	0.15	The business contract sector of one Brazilian health institute
	**Total**		**10.31**	

[1]Brazil. Diário Oficial do Município Lei nº 10.948, 13 de julho de 2016. Available: http://portal6.pbh.gov.br/dom/iniciaEdicao.do?method=DetalheArtigo&pk=1165759; [2]Brazil. Ministry of Health. SIGTAP—Sistema de Gerenciamento da Tabela de Procedimentos, Medicamentos e OPM do SUS. 2020. Available: http://sigtap.datasus.gov.br/tabela-unificada/app/sec/inicio.jsp.
